# The interaction between tobacco use and oral health among tribes in central India

**DOI:** 10.1186/1617-9625-10-16

**Published:** 2012-10-19

**Authors:** Sunali Khanna

**Affiliations:** 1 Nair Hospital Dental College, Maharashtra University of Health Sciences, Mumbai, 400008, India

**Keywords:** Tobacco related practices, Central India, Oral precancer

## Abstract

**Background:**

A study was undertaken to evaluate the effect of tobacco related practices on oral health of tribes in Central India. The use of smokeless tobacco, gutkha & associated products is on the rise amongst the younger generation making oral precancer & cancer a public health concern.

**Methodology:**

A pioneering study was conducted to evaluate the tobacco related practices amongst tribes and its impact on oral health. The study included 411 tribals of the Baiga group. Guided dialogue techniques and proforma based evaluation formed a part of the study.

**Result:**

53.04% of individuals between 21 to 40yrs are addicted to deleterious habits. There is a marked consumption (72%) of tobacco & associated products among the geriatric population (60 yrs & above).Insecure livelihoods, malnutrition & increased stress levels contribute to the stark increase of addiction of tobacco related practices.

**Conclusion:**

The healthcare infrastructure needs to be upgraded to meet the demands of changing disease profile amongst the vulnerable population. Assessment of impact of disease on existing public health would enable formulation of adaptive measures and suggestions for amelioration.

## Background

In South-central Asia 80% of head and neck cancers are found in the oral cavity and oropharynx. Oral squamous cell carcinoma comprises over 90% of the malignancies, which begin as inflammatory lesions such as leukoplakia, erythroplakia and erythroleukoplakia [1,2]. Notably, oral cancer is one of the few cancers whose survival rate has not improved over 30 years, while during the past three decades a 60% increase in oral cancer in adults under the age of 40 has been documented [3,4]. 

**Figure 1:**
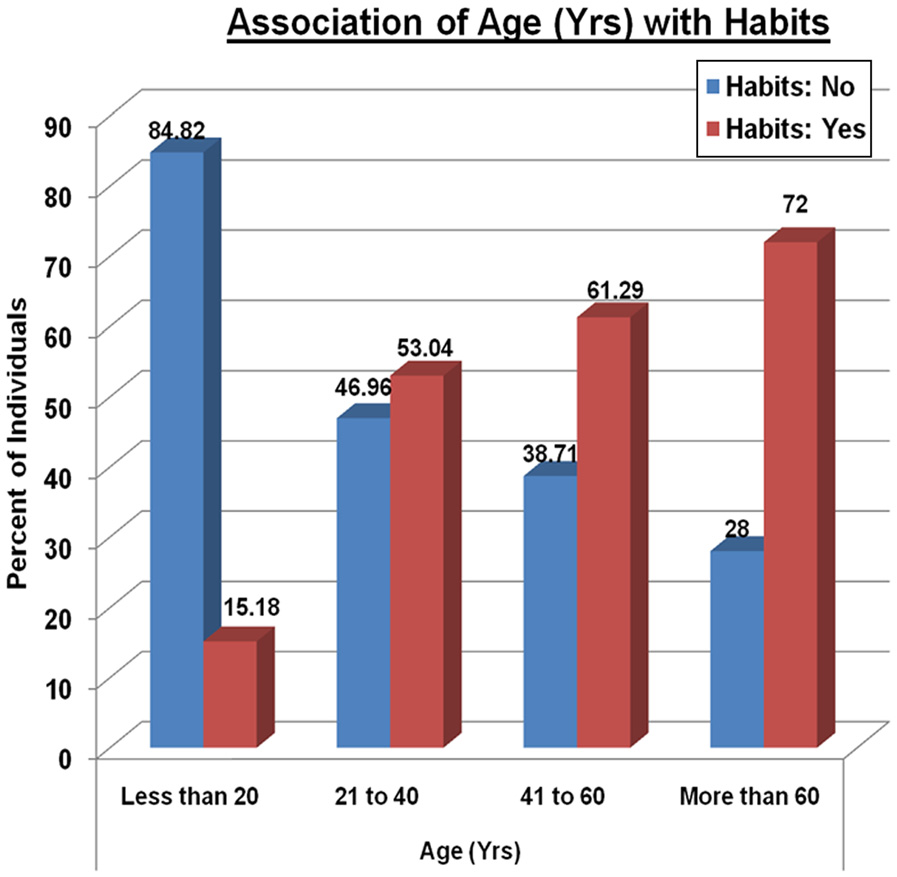
The distribution of tobacco use throughout age categories among tribal members in Central India.

Oral pre-cancer (Oral Submucous Fibrosis (OSMF) & Leukoplakia) and cancers are caused by gene environment interactions, and large consumption of tobacco and/or areca nut amongst other factors [5,6]. In the area under study, “Tambaku” is the most commonly consumed form of smokeless tobacco. Smokeless forms of tobacco, due to their placement in the oral cavity, increase the concentration of carcinogens and the failure to clean the carcinogens from the surface, augment this effect. Tobacco when added to areca nut, lime, flavouring agents and catechu is termed “Gutkha”; which is a commercially available product within India.

With the above points in mind, the aim was to evaluate the impact of tobacco use on the oral health of the Baiga tribes of Madhya Pradesh, in Central India.

## Methods

The study included 411 Baiga tribals, data was collected during field visits to Dhoba, Ranchha, Saraswahi, and Barbaspur villages of block Manpur, India. The approval of the District Administration and the regional Chief Medical & Health authority was obtained. Informed consent was taken prior to the study from the tribals. Tribals, traditional healers & health care professionals were interviewed with the use of questionnaires and a clinical examination was performed during which the presence of leukoplakia, OSMF and burning mouth syndrome were evaluated by assessing the symptoms & signs, after systematic recording of the history of each respondent [7-10].

## Results & discussion

Our study population’s age ranged between 11 – 85 years. Out of the total study population, 233 were males while 178 were females. There were 162 who were classified as Illiterate, 20 had a primary level of education, 157 had completed Secondary education and 72 had a Higher Secondary level of education and above. The extensive use of tobacco can be attributed to lack of awareness.

The data revealed that 53.0-% of the total population aged 21–40 years and 15.2% of tribals less than 20yrs of age currently used tobacco (smoked & smokeless) & tobacco associated products (gutkha, areca nut, etc.) Moreover, 61.3% of individuals between 41-60yrs used tobacco products, while a marked rise in consumption of tobacco & associated products was seen amongst 72% of the geriatric population (60 yrs. & above) (Figure 1). This association with age was significant (p value <0.01). In the population assessed the prevalence of oral disabilities seen were - leukoplakia (10.7%), OSMF (6.3%) and Burning Mouth Syndrome (11.4%); which may be attributed to the tobacco related habits. The gender- based comparison revealed that 4% of the total female population & 23% of the total male population had leukoplakia (p value <0.01). Moreover, 25% of tribals who developed leukoplakia used tobacco as compared to 0.42% of tribals who had leukoplakia but did not use tobacco. Finally, 28.8% of tribals with OSMF reported gutkha consumption in comparison to 3.06% who had OSMF but no associated tobacco related habit.

## Conclusion

This study aimed to assess the tobacco related practices and related oral health amongst the Baiga tribals in Central India. The higher prevalence of tobacco use (smokeless and smoked) and associated tobacco product consumption by the younger population is an issue of significant concern. This study further revealed that tobacco use amongst Baiga tribals of Central India had a serious impact on their oral health status. Working towards the mitigation of factors affecting tobacco menace at the individual level as well as at the community level should be implemented as a part of a long term commitment to safeguard public health. Anti- tobacco initiatives are thus warranted [11]. This is a pioneering attempt at the grass root level in Central India and points to a holistic approach that requires the integrating efforts of multidisciplinary teams, if tobacco use is going to be combated using a decentralized approach.

## Competing interests

The author declares that there is no competing interest.
